# Longitudinal follow-up by MR angiography reveals progressive dilatation of the distal aorta after aortic root replacement in Marfan syndrome

**DOI:** 10.1007/s00330-023-09684-z

**Published:** 2023-05-09

**Authors:** Alexander Lenz, Malte Warncke, Felicia Wright, Julius Matthias Weinrich, Bjoern P. Schoennagel, Frank Oliver Henes, Gerhard Adam, Yskert von Kodolitsch, Gerhard Schoen, Peter Bannas

**Affiliations:** 1 Department of Diagnostic and Interventional Radiology and Nuclear Medicine, University Medical Center Hamburg-Eppendorf, Martinistr. 52, 20246 Hamburg, Germany; 2 Department of Diagnostic and Interventional Radiology, BG Hospital Hamburg, Hamburg, Germany; 3 Department of Cardiovascular Medicine, University Heart and Vascular Center Hamburg, Hamburg, Germany; 4 Institute of Medical Biometry and Epidemiology, University Medical Center Hamburg-Eppendorf, Hamburg, Germany

**Keywords:** Aorta, thoracic, Aortic aneurysm, Heart defects, congenital, Marfan syndrome, Magnetic resonance angiography

## Abstract

**Objectives:**

To define and compare growth rates of the distal aorta in Marfan patients with and without aortic root replacement using serial MR angiography (MRA).

**Methods:**

We retrospectively included 136 Marfan patients with a total of 645 MRAs who underwent a median of five MRAs (range: 2–13) at 1.5 T and 3 T in annual intervals. Of these, 41 patients (34.8 ± 12 years) had undergone aortic root replacement. The remaining 95 patients (29.0 ± 17 years) still had a native aorta and served as the control group. Thoracic aortic diameters were independently measured at eleven predefined levels. Estimated growth rates were calculated using a mixed effects model adjusted for sex, age, BMI, and medication.

**Results:**

Marfan patients with aortic root replacement revealed the highest mean estimated growth rate in the proximal descending aorta (0.77 mm/year, CI: 0.31–1.21). Mean growth rates at all levels of the distal thoracic aorta were significantly higher in patients with aortic root replacement (0.28–0.77 mm/year) when compared to patients without aortic root replacement (0.03–0.07 mm/year) (all *p* < 0.001). Antihypertensive medication, gender, and BMI had no significant impact on the distal aortic growth rates.

**Conclusion:**

Distal thoracic aortic diameters increase at a significantly higher rate in Marfan patients with aortic root replacement compared to Marfan patients without aortic root replacement. Further studies are warranted to investigate if the increased growth rate of the distal thoracic aorta after aortic root replacement is caused by altered hemodynamics due to the rigid aortic root graft or due to the general genetic disposition of post-operative Marfan patients.

**Clinical relevance statement:**

High growth rates of the distal aorta after aortic root replacement underline the need for careful life-long aortic imaging of Marfan patients after aortic root replacement.

**Key Points:**

• *Aortic growth rates in Marfan patients with aortic root replacement are highest in the mid-aortic arch, the proximal- and mid-descending aorta.*

• *Growth rates of the distal thoracic aorta are significantly higher in Marfan patients with aortic root replacement compared to Marfan patients without aortic root replacement.*

• *Antihypertensive medication, gender, and BMI have no significant impact on distal aortic growth rates in Marfan patients.*

**Supplementary Information:**

The online version contains supplementary material available at 10.1007/s00330-023-09684-z.

## Introduction

Marfan syndrome is a genetic disorder of connective tissue caused by mutations in the Fibrilin-1 (*FBN1)* gene encoding the matrix protein fibrilin-1 [1]. The disease affects different parts of the human body, including the heart and blood vessels [2–4]. The most frequent cardiovascular complication in Marfan patients is progressive aortic root dilatation [1, 5]. Aortic root aneurysms increase the risk of lethal aortic dissection [1, 5–7]. An absolute aortic diameter of 50 mm, or 45 mm in patients who have additional risk factors, or a rapid increase of aortic root diameters (≥ 5 mm/year) indicates elective prophylactic aortic surgery in Marfan patients to mitigate the risk of aortic dissection [8, 9]. The surgical therapy of proximal aortic root disease is now well established with excellent long-term survival of post-operative Marfan patients [10, 11].

However, despite prolonged survival, aortic root replacement has led to an increased number of Marfan patients who experience aortic complications beyond the replaced aortic root [12, 13]. The progression of distal aortic diameters has been described in Marfan patients after aortic root replacement [14–16].

Precise growth rates of the distal aorta have not been defined for Marfan patients after aortic root replacement. MR angiography enables precise monitoring of aortic diameters in Marfan patients since it allows accurate visualization of aortic morphology at all levels of the thoracic aorta [17–21].

Therefore, the aim of this study was to define and compare growth rates of the distal aorta in Marfan patients with and without aortic root replacement using serial MR angiography.

## Materials and methods

### Study sample

The local institutional review board approved our retrospective single-center study and waived the requirement for written informed consent.

Patients with confirmed Marfan syndrome, who were referred for routine MR angiography between August 2006 and February 2020 were screened retrospectively for inclusion in the study. Marfan diagnosis was established according to the latest Ghent nosology and confirmed by genetic analyses with the sequencing of the *FBN1* gene [9].

Elective replacement of the aortic root was performed in Marfan patients with a maximum diameter of ≥ 50 mm, or ≥ 45 mm in patients who have additional risk factors, or rapid increase of aortic root diameters (≥ 5 mm/year), as recommended by the ESC guideline [9]. In our clinic, Marfan patients eligible for prophylactic aortic surgery are discussed in a multidisciplinary board (cardiology, cardiac surgery, radiology) before surgery is proposed. The proposal is then discussed with the patients. As a result, > 90% of Marfan patients eligible for prophylactic aortic root replacement undergo surgery.

Patients were included in the study if they had (i) confirmed diagnosis of Marfan syndrome, ii) at least two MR angiography studies to allow for calculation of growth rates, (iii) and at least one pre-operative MRI study in case of prior aortic surgery.

Patients were excluded from the study if they had persistent residual dissection distal to the aortic graft, which might alter natural aortic growth rates.

All patient records were screened for the date of aortic surgery and/or complications after surgery.

### MR angiography

MR angiography (MRA) of the entire aorta was performed with or without [22] contrast enhancement as described previously using either 1.5 [19, 23] or 3-T [24] MR systems equipped with multi-channel receiver coils for cardiovascular imaging (Achieva and Ingenia, Philips Medical Systems).

### Image analyses

**Fig. 1:**
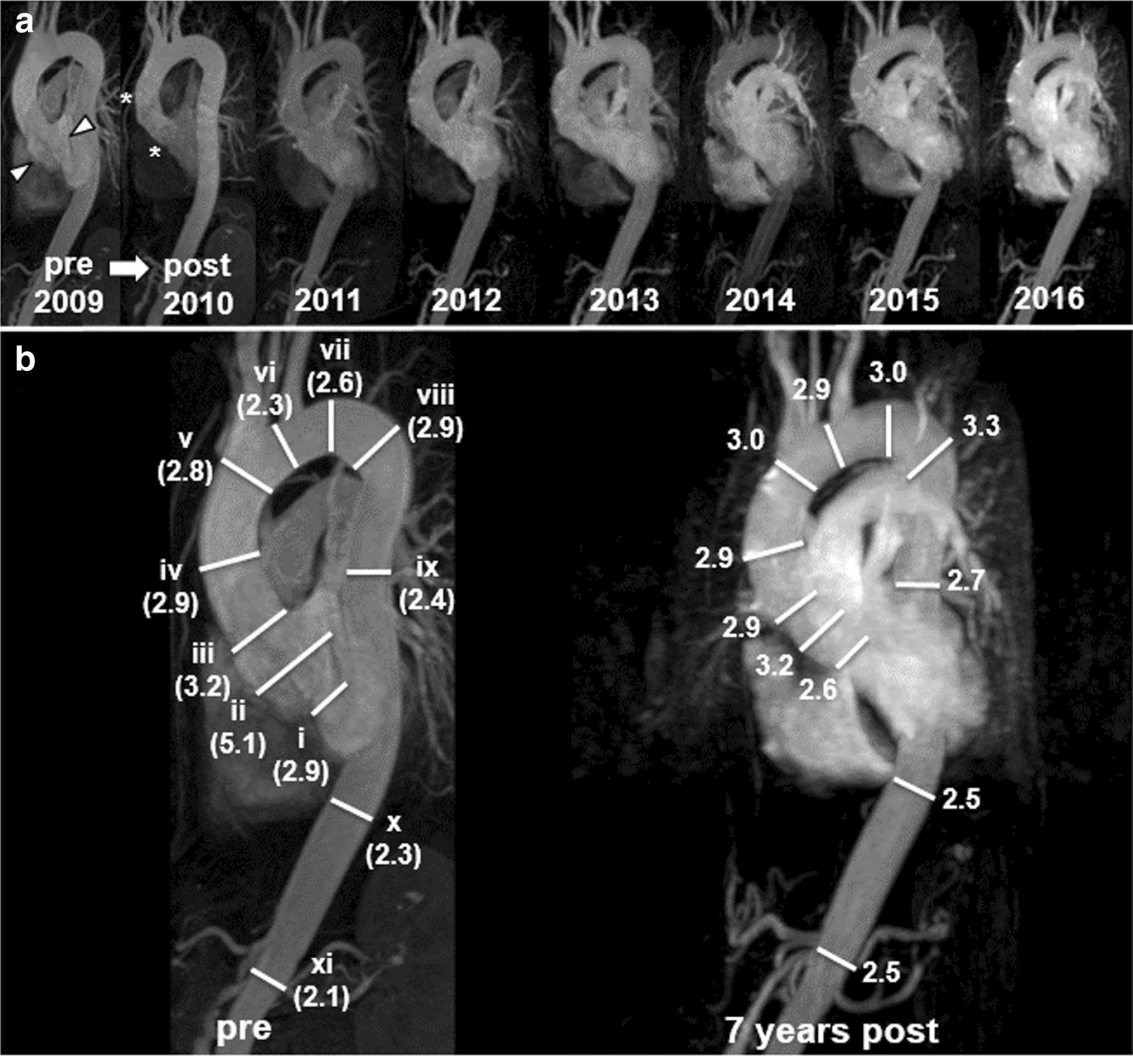
MRA-based long-term follow-up of the thoracic aorta after elective aortic root replacement. **a** Annual MRAs of a 59-year-old woman with Marfan syndrome without and with aortic root replacement (David procedure) between 2009 and 2016. Pre-operative (pre) MRA in 2009 demonstrates an aortic root aneurysm of 5.1 cm (arrowheads). Post-operative (post) MRAs demonstrate a constant diameter of the aortic root graft (asterisks) but continuously increasing diameter of the distal aorta (2010–2016). **b** Side-by-side comparison of pre-operative and post-operative follow-up MRA after seven years demonstrate increasing diameters (indicated in cm) at all measured levels of the distal aorta. White lines indicate the eleven measurement levels. From proximal to distal: (i) aortic annulus, (ii) sinuses of Valsalva, (iii) sinotubular junction, (iv) mid-ascending aorta, (v) proximal aortic arch, (vi) mid-aortic arch, (vii) proximal descending thoracic aorta (2 cm distal to left subclavian artery), (viii) proximal descending thoracic aorta at maximum diameter, (ix) mid-descending aorta, (x) diaphragmatic aorta, (xi) abdominal aorta. The aortic root graft was measured at the same levels as the pre-operative ascending aorta for better comparison and identically numbered measurement levels before and after aortic root replacement

All MR images were interpreted on state-of-the-art RIS/PACS workstations (Centricity™ RIS-i 7, GE General Electric Company). Two radiologists with 6 and 4 years of experience in cardiovascular imaging performed all diameter measurements individually from the inner-to-inner edge of the aortic wall at the following eleven aortic levels: aortic annulus, sinuses of Valsalva, sinotubular junction, mid-ascending aorta, proximal aortic arch, mid-aortic arch, proximal descending thoracic aorta (2 cm distal to the left subclavian artery), proximal descending thoracic aorta at maximum diameter, mid-descending aorta, diaphragmatic aorta and abdominal aorta (Fig. 1) [21, 25].

Diameter measurements were performed perpendicular to the blood-filled lumen at all levels on identically orientated para-sagittal MRA images [26]. Using identically oriented para-sagittal images avoided possible user influence introduced by individually performed multiplanar reformations [19, 23, 27]. Readers were free to choose appropriate slices displaying the maximal profile of the aorta from the stacks of para-sagittal images.

### Statistical analyses

Inter-rater reliability of aortic diameter measurements was evaluated. Further statistical analyses were based on the calculated mean of measurements of both readers. Intraclass correlation coefficients (ICC) were calculated based on a two-way mixed effects model (single rater, absolute agreement).

The Shapiro–Wilk test was used to evaluate whether the parameters were normally distributed. Data were compared by a two-sided paired t-test if normally distributed and by the Mann–Whitney test if non-normally distributed.

We have performed a locally weighted polynomial regression (LOESS) to check the linearity of the slope (data not shown). Growth rates at different aortic levels were calculated in all Marfan patients by applying a linear mixed model to describe changes in diameter.

Results were reported as estimated marginal means, which are represented in graphs with their corresponding 95%-confidence intervals (CIs). A multiple linear mixed regression model was used to adjust for age, gender, BMI, and antihypertensive medication between Marfan patients with and without aortic root replacement. *p* values < 0.05 were considered a statistically significant difference. Statistical analyses were computed by a biomedical statistics expert using R (R Core Team. R: A Language and Environment for Statistical Computing. R Foundation for Statistical Computing, 2021) [28].

## Results

### Study population

Within the study period, 362 patients with confirmed Marfan syndrome were treated in our institution.

We included 136 Marfan patients (37.6%, 58 men; 78 women, age range 7–71 years; mean age 30.7 ± 16 years, BMI: 22.1 ± 4.9 kg/m^2^) with a total of 645 MRAs. The median number of MRA scans was five (range: 2–13). The time interval between individual scans was 18.2 ± 9.6 months. The median monitoring time was 5.8 years (range 0.6–13.2 years) (Table 1).

**Table 1:** Demographics of 136 Marfan patients with and without aortic root replacement and number of MRAs

	Total (*n* = 136)	Without surgery (*n* = 95)	With surgery (*n* = 41)	*p* values
Sex, female; *n* (%)	78 (57)	61 (64.2)	17 (41.5)	**0.02**
Antihypertensive medication; *n* (%)	91 (67)	57 (60)	34 (82.9)	**0.01**
Mean age ± SD at first measurement; years	30.7 ± 16	29 ± 17	34.8 ± 12	**0.01**
Mean BMI ± SD at first measurement; kg/m^2^	22.1 ± 4.9	21.7 ± 5.2	23.2 ± 3.7	0.06
Median number of MRAs (range)	5 (2–13)	4 (2–12)	5 (2–13)	0.05
Median monitoring time; years (range)	5.8 (0.6–13.2)	5.2 (0.9–10.9)	6 (0.6–13.2)	0.15
Mean time interval ± SD between MRAs; months	18.2 ± 9.6	18.8 ± 9.9	16.9 ± 8.9	**0.02**

Data were compared by a two-sided paired t-test if normally distributed and by the Mann–Whitney test if non-normally distributed. *p* < 0.05 indicates a statistically significant difference. Significant values are in boldface

We excluded 226 patients (62.4%). Of these, 214/226 patients (94.7%) were excluded due to the lack of at least two MR angiography studies precluding the estimation of growth rates. We further excluded 12/226 patients (5.3%) with persistent residual dissection distal to the aortic graft, which might alter natural aortic growth rates.

Forty-one of the included patients (24 men; 17 women; mean age: 34.8 ± 12 years, BMI: 23.2 ± 3.7 kg/m^2^, antihypertensive medication: 82.9%) underwent elective aortic root replacement during this period due to increased aortic root diameter or rapid growth rate. David’s procedure (valve-sparing aortic root replacement) was performed in 37 patients and the Bentall procedure (aortic root and aortic valve replacement) was performed in four patients. The median number of MRA scans was five (range: 2–13) (Table 1).

The remaining 95 patients had no surgical interventions (34 men; 61 women; mean age: 29.0 ± 17 years, BMI: 21.7 ± 5.2 kg/m^2^, antihypertensive medication: 60%). The median number of MRA scans was four (range: 2–12) (Table 1).

### Inter-rater reliability of aortic diameter measurements

A high degree of reliability was found between both readers for diameter measurements at all aortic levels. The ICC was between 0.79 and 0.87.

### Aortic diameters of Marfan patients with and without aortic root replacement

Mean diameters at baseline and last follow-up in Marfan patients with and without aortic root replacement are displayed in Table 2. In Marfan patients with aortic root replacement, a maximal increase of 3 mm was observed for the maximal diameter of the proximal descending aorta with an increase from 2.52 ± 0.37 to 2.81 ± 0.40 cm. In Marfan patients without aortic surgery, the maximal increase of 11 mm was observed at the level of the sinuses of Valsalva with an increase from 3.67 ± 0.58 to 3.78 ± 0.59 cm. The distal aorta diameters showed less increase in absolute aortic diameters.

**Table 2:** Aortic diameters of Marfan patients with and without aortic root replacement at first and last follow-up MRA

	Without surgery (*n* = 95)		With surgery (*n* = 41)	
	Baseline (cm)	Last follow-up (cm)	Baseline (cm)	Last follow-up (cm)
Aortic annulus	2.35 ± 0.35	2.41 ± 0.36	2.64 ± 0.33	2.66 ± 0.32
Sinuses of Valsalva	3.67 ± 0.58	3.78 ± 0.59	3.40 ± 0.36	3.41 ± 0.37
Sinotubular junction	2.78 ± 0.48	2.85 ± 0.48	2.84 ± 0.35	2.86 ± 0.34
Mid-ascending aorta	2.67 ± 0.49	2.72 ± 0.51	2.79 ± 0.40	2.82 ± 0.40
Proximal aortic arch	2.48 ± 0.45	2.52 ± 0.45	2.57 ± 0.37	2.76 ± 0.37
Mid-aortic arch	2.17 ± 0.37	2.21 ± 0.37	2.19 ± 0.26	2.45 ± 0.30
Proximal descending aorta 1	2.12 ± 0.39	2.16 ± 0.39	2.22 ± 0.33	2.51 ± 0.34
Proximal descending aorta 2	2.23 ± 0.42	2.27 ± 0.43	2.52 ± 0.37	2.81 ± 0.40
Mid-descending aorta	2.09 ± 0.36	2.12 ± 0.37	2.20 ± 0.30	2.51 ± 0.30
Diaphragmatic aorta	1.90 ± 0.36	1.92 ± 0.35	1.95 ± 0.27	2.13 ± 0.36
Abdominal aorta	1.83 ± 0.36	1.85 ± 0.36	1.88 ± 0.26	2.06 ± 0.25

Values represent mean ± SD, unless otherwise indicated

### Aortic growth rates of Marfan patients with and without aortic root replacement

**Fig. 2:**
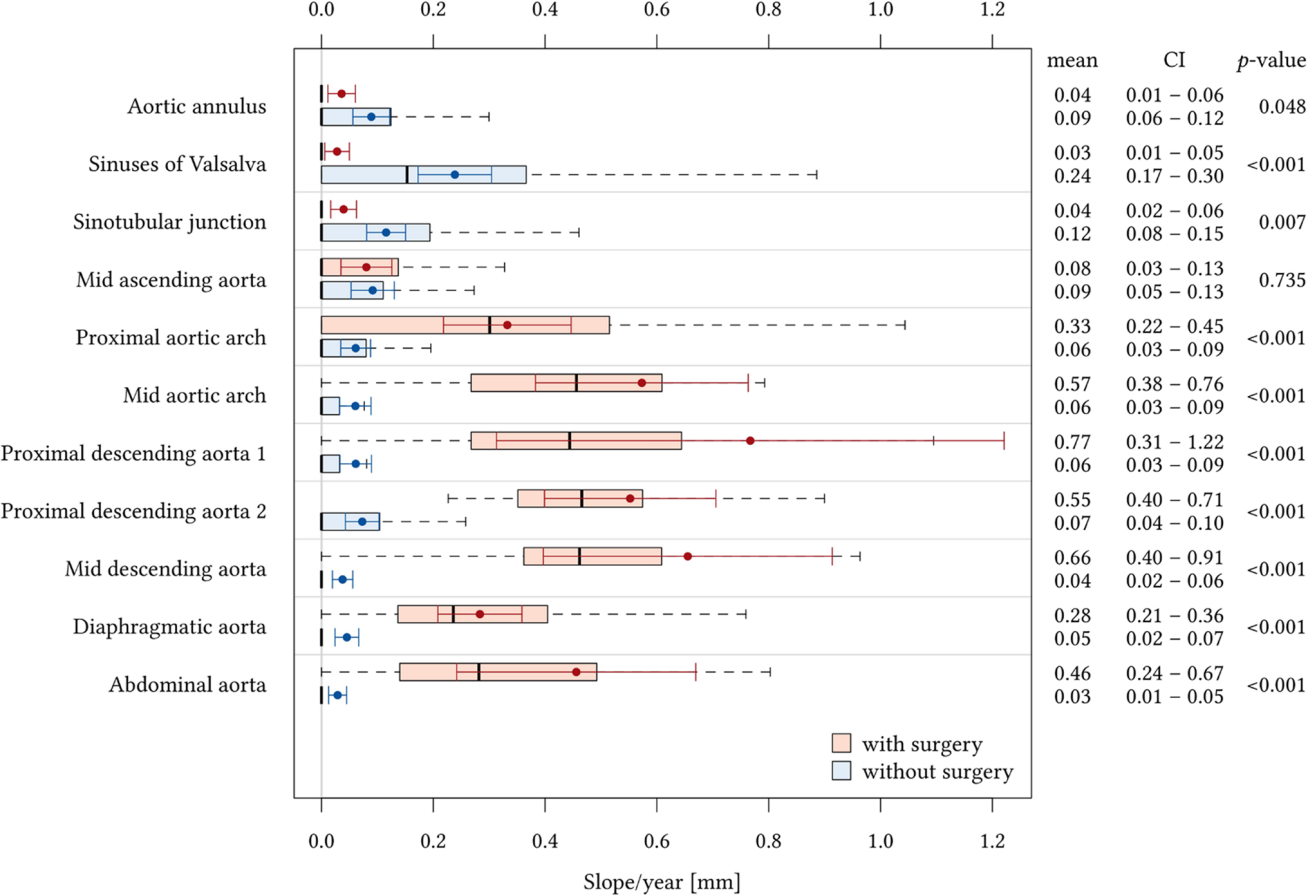
Comparison of aortic growth rates in Marfan patients with and without aortic root replacement. Boxplots indicate median growth rates by thick vertical black lines and interquartile ranges for each aortic level in Marfan patients with (red) and without aortic root replacement (blue). Red and blue dots denote the means and lines denote the corresponding confidence intervals, as the figures on the right margin. *p* values derived from the t-test of the pairwise comparisons of the aortic levels. The highest aortic growth rates in patients with aortic root replacement were observed in the mid-aortic arch, the proximal and mid-descending aorta. Significantly higher growth rates at all levels distal to the aortic root graft were observed in Marfan patients with aortic root replacement when compared to Marfan patients without aortic root replacement (all *p* < 0.001). Arithmetic means and confidence intervals are displayed by dots and solid horizontal lines. Boxes indicate interquartile ranges and whiskers indicate upper quartiles. Ranges are indicated by dashed lines. Outliers (1.5xIQR) are excluded from this graph. Numbers indicate mean growth rates and confidence intervals (mm/year)

In Marfan patients with aortic root replacement, the highest aortic growth rates of the distal aorta were observed in the mid-aortic arch (0.57 mm/year, CI: 0.38–0.76 mm/year), proximal descending aorta (0.77 mm/year, CI: 0.31–1.22 mm/year), and mid-descending aorta (0.66 mm/year, CI: 0.40–0.91 mm/year) (Fig. 2). The lowest growth rates of the distal aorta were observed at the level of the diaphragmatic aorta (0.28 mm/year, CI: 0.21–0.36 mm/year) and the abdominal aorta (0.46 mm/year, CI: 0.24–0.67 mm/year). As expected, the diameter of the aortic root graft showed little if any growth.

In Marfan patients without aortic root replacement, the highest aortic growth rates were observed for the sinuses of Valsalva (0.24 mm/year, CI: 0.17–0.30 mm/year) and for the sinotubular junction (0.12 mm/year, CI: 0.08–0.15 mm/year). Low growth rates were observed for all levels of the distal aorta.

Comparison of Marfan patients with and without aortic root replacement revealed significantly higher growth rates of aortic diameters at all levels from the proximal aortic arch to the abdominal aorta for Marfan patients with aortic root replacement (all *p* < 0.001).

At the mid-ascending aorta, no significant difference in growth rates was observed between Marfan patients with (0.08 mm/year, CI: 0.03–0.13 mm/year) and without (0.09 mm/year, CI: 0.05–0.13 mm/year) aortic root replacement (*p* = 0.735).

Growth rates for the ascending aorta at the levels of the aortic anulus, sinuses of Valsalva, and sinotubular junction were significantly higher in Marfan patients without aortic root surgery when compared to Marfan patients with aortic root surgery, where measurements were made at corresponding levels of the graft.

### Linear mixed regression analysis

**Fig. 3:**
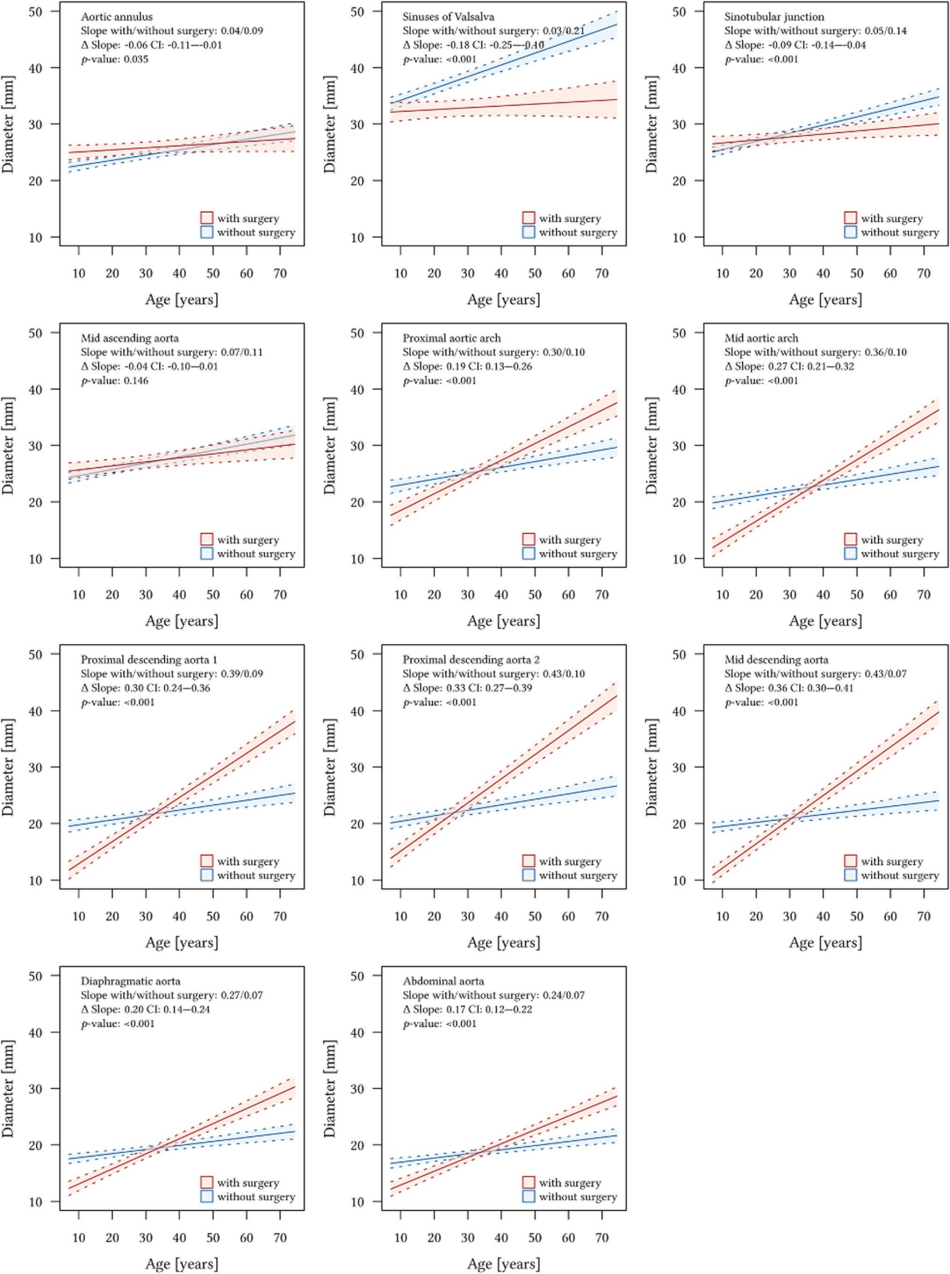
Multiple regression models for age and diameters at all aortic levels in Marfan patients with (red) and without (blue) aortic root replacement with 95%-confidence intervals. Slopes (mm/year). Adjusted for medication, sex, and BMI. The multiple regression models demonstrated significantly higher growth rates distal to the aortic root graft in patients with surgery compared to patients without surgery (*p* < 0.001 for all anatomical levels). The highest slope differences were observed at the proximal descending thoracic aorta (0.30 mm/year, *p* < 0.001), the proximal descending thoracic aorta at the maximum diameter (0.33 mm/year, *p* < 0.001), and the mid- descending aorta (0.36 mm/year, *p* < 0.001)

Multiple linear mixed regression adjusted for age, sex, BMI, and antihypertensive medication—performed for all 11 aortic levels—confirmed that aortic root replacement is an independent predictor for increased aortic growth rates distal to the aortic root graft in Marfan patients (all *p* < 0.001). The highest growth rate differences between Marfan patients with and without aortic root replacement were observed for the proximal descending aorta two cm distal to the left subclavian artery (0.30 mm/year, CI: 0.24–0.36 mm/year; *p* < 0.001), the proximal descending aorta at the maximum diameter (0.33 mm/year, CI: 0.27–0.39 mm/year; *p* < 0.001) and the mid-descending aorta (0.36 mm/year, CI: 0.30–0.41 mm/year; *p* < 0.001) (Fig. 3, Appendix).

## Discussion

Our MRA study reveals that distal thoracic aortic diameters increase at a significantly higher rate in Marfan patients with aortic root replacement when compared to Marfan patients without aortic root replacement.

The differences in aortic growth rates were dependent on the different aortic levels. Growth rates for the ascending aorta at the levels of the aortic anulus, sinuses of Valsalva, and sinotubular junction were significantly higher in Marfan patients without aortic root surgery when compared to Marfan patients with aortic root surgery. This is an expected result since no growth is present along the rigid aortic graft in post-operative Marfan patients. However, continued growth takes place in the native ascending aorta of pre-operative Marfan patients [5, 29, 30].

At the mid-ascending aorta, no significant difference in growth rates was observed between Marfan patients with and without aortic root replacement. This aortic level shows less growth when compared to proximal aortic levels of pre-operative patients. This part of the aorta is therefore not always replaced by the graft, which is fitted individually, explaining the comparable growth rates of pre- and post-operative Marfan patients at this level.

Growth rates of aortic diameters for all levels from the proximal aortic arch to the abdominal aorta were significantly higher for Marfan patients with aortic root replacement when compared to Marfan patients without aortic surgery. There are two possible explanations for this observation.

The first possible explanation may be that the replacement of the aortic root by a rigid graft leads to increased velocities and wall shear stress in the aortic arch and the descending aorta resulting in progressive dilatation. Further studies using modern imaging techniques such as 4D flow MRI are warranted [21, 31]. Comparative analyses of Marfan patients with and without aortic root replacement using 4D flow MRI will allow a comparison of three-dimensional blood flow profiles and quantification of flow velocity, flow eccentricity, and wall shear stress in the distal aorta.

The second possible explanation of the increased growth rate of the distal aorta after aortic root replacement might be the general genetic disposition of the cohort of post-operative Marfan patients, which might be different in the pre-operative cohort. Not all Marfan patients develop aortic root aneurysms or only later in life [32]. Therefore, the pre-operative cohort of Marfan patients in our study is likely to be a mix of patients with low and high growth rates, which is indicated by the relatively high 95% confidence intervals at the level of the sinuses of Valsalva. On the contrary, the post-operative cohort is likely to consist of Marfan patients with high growth rates, which is underlined by the fact that these patients had to undergo preventive surgery on their dilatated aorta.

This notion is supported by the fact that maximal growth rates of the distal aorta (0.77 mm/year, CI: 0.31–1.22 mm/year) in Marfan patients *after* aortic root surgery were higher than maximal growth rates of the mid-ascending aorta (0.24 mm/year, CI: 0.17–0.30 mm/year) in Marfan patients *before* aortic root surgery.

The defined high growth rates of the distal aorta using MRA is an important observation with clinical implications. Our results underline the need for careful life-long aortic imaging of Marfan patients after aortic root replacement, as recommended by current guidelines: Marfan patients who have undergone aortic root replacement should undergo annual surveillance by MRI (or CT) to evaluate changes in the distal aorta. If normal in diameter and unchanged after two years, surveillance imaging may be performed every other year [33].

Previous studies have shown that the prolonged survival of Marfan patients after aortic surgery has led to an increase in aortic complications beyond the root [13–15]. We therefore recommend non-contrast MRA techniques [23] providing high image quality, precise and reproducible aortic diameter measurements with high diagnostic performance in the detection of relevant aortic pathologies in Marfan patients after aortic root replacement [13–15]. CT angiography may be used as an alternative imaging technique when MRA is contraindicated [34].

The major limitation of our study is its retrospective nature precluding to elucidate the exact underlying cause for the increased growth rates of the distal aorta in post-operative Marfan patients. Further prospective and longitudinal follow-up studies using modern MR imaging techniques such as 4D flow MRI are needed to determine the cause of the increased growth rates of the distal aorta in post-operative Marfan patients.

## Conclusion

Distal thoracic aortic diameters increase at a significantly higher rate in Marfan patients with aortic root replacement compared to Marfan patients without aortic root replacement. Further studies are warranted to investigate if the increased growth rate of the distal aorta after aortic root replacement is caused by altered hemodynamics due to the rigid aortic root graft or due to the general genetic disposition of post-operative Marfan patients.

### Supplementary Information

Below is the link to the electronic supplementary material.

Supplementary file1 (PDF 69 KB)

## Abbreviations

FBN1: Fibrillin-1
ICC: Intraclass correlation coefficients
MRA: Magnetic resonance angiography

## Declarations

### Guarantor

The scientific guarantor of this publication is A. Lenz.

### Statistics and biometry

One of the authors, G. Schoen, is an expert in Medical Biometry and has significant statistical expertise.

### Study subjects or cohorts overlap

No study subjects or cohorts have been previously reported.

### Methodology

• retrospective

• observational

• performed at one institution
