# Supplementary material for: Longitudinal follow-up by MR angiography reveals progressive dilatation of the distal aorta after aortic root replacement in Marfan syndrome
Source: Eur Radiol. 2023 May 9;33(10):6984–92. doi: 10.1007/s00330-023-09684-z (PMC10511572; doi:10.1007/s00330-023-09684-z)
Supplement: Supplementary file 1 — Supplementary file1 (PDF 69 KB) [file 330_2023_9684_MOESM1_ESM.pdf]

ELECTRONIC SUPPLEMENTARY MATERIAL

Longitudinal follow-up by MR angiography reveals progressive dilatation of the distal aorta after aortic root replacement in Marfan syndrome

Appendix: Detailed results for the multiple regression models adjusted for medication, sex and BMI.

Groups	Effects	*Estimated Coefficient*	*95% CI*	*p-value*
**Annulus**	Age at measurement	0.093	0.064, 0.122	<0.001
	Group: with surgery	2.963	1.149, 4.769	0.002
	Antihypertensive medication: yes	0.296	-0.250, 0.852	0.291
	Sex: male	2.970	1.843, 4.094	<0.001
	BMI	0.078	0.033, 0.122	<0.001
	Age at measurement x Group: with surgery	-0.056	-0.108, -0,005	0.0035
**Sinuses of Valsalva**	Age at measurement	0.209	0.168, 0.250	<0.001
	Group: with surgery	-0.235	-2.644, 2.149	0.848
	Antihypertensive medication: yes	0.360	-0.407, 1.139	0.356
	Sex: male	3.348	1.742, 4.950	<0.001
	BMI	0.128	0.066, 0.190	<0.001
	Age at measurement x Group: with surgery	-0.176	-0.247, -0.105	<0.001
**Sinutubular junction**	Age at measurement	0.145	0.117, 0.174	<0.001
	Group: with surgery	2.094	0.283, 3.900	0.026
	Antihypertensive medication: yes	0.221	-0.452, 0.899	0.519
	Sex: male	2.597	1.562, 3.633	<0.001
	BMI	0.059	0.005, 0.116	0.034
	Age at measurement x Group: with surgery	-0.092	-0.141, -0.044	<0.001
**Mid-ascending aorta**	Age at measurement	0.112	0.080, 0.144	<0.001
	Group: with surgery	1.451	-0.532, 3.428	0.155
	Antihypertensive medication: yes	-0.127	-0.801, 0.543	0.712
	Sex: male	2.540	1.260, 3.820	<0.001
	BMI	0.020	-0.034, 0.077	0.469
	Age at measurement x Group: with surgery	-0.041	-0.096, 0.014	0.146
**Proximal aortic arch**	Age at measurement	0.104	0.068, 0.141	<0.001
	Group: with surgery	-6.437	-8.933, -3.896	<0.001
	Antihypertensive medication: yes	0.343	-0.508, 1.171	0.419
	Sex: male	1.554	0.322, 2.786	0.016
	BMI	0.018	-0.051, 0.090	0.615
	Age at measurement x Group: with	0.193	0.128, 0.255	<0.001

	surgery			
**Mid-aortic arch**	Age at measurement	0.096	0.063, 0.129	<0.001
	Group: with surgery	-9.811	-12.030, -7.514	<0.001
	Antihypertensive medication: yes	-0.092	-0.895, 0.704	0.823
	Sex: male	1.046	-0.069, 2.158	0.071
	BMI	0.048	-0.018, 0.116	0.159
	Age at measurement x Group: with surgery	0.267	0.206, 0.324	<0.001
**Prox. descending thoracic aorta 1**	Age at measurement	0.087	0.055, 0.120	<0.001
	Group: with surgery	-9.932	-12.180, -7.599	<0.001
	Antihypertensive medication: yes	0.414	-0.431, 1.231	0.326
	Sex: male	0.939	-0.264, 2.139	0.133
	BMI	0.047	-0.019, 0.116	0.165
	Age at measurement x Group: with surgery	0.304	0.244, 0.361	<0.001
**Prox. descending thoracic aorta 2**	Age at measurement	0.097	0.065, 0.130	<0.001
	Group: with surgery	-8.589	-10.736, -6.423	<0.001
	Antihypertensive medication: yes	-0.137	-0.900, 0.621	0.724
	Sex: male	1.419	0.043, 2.796	0.047
	BMI	0.037	-0.023, 0.100	0.231
	Age at measurement x Group: with surgery	0.330	0.272, 0.387	<0.001
**Mid-descending aorta**	Age at measurement	0.071	0.041, 0.101	<0.001
	Group: with surgery	-10.947	-12.822, -8.989	<0.001
	Antihypertensive medication: yes	-0.210	-1.035, 0.610	0.617
	Sex: male	1.075	-0.117, 2.266	0.078
	BMI	0.077	0.010, 0.145	0.023
	Age at measurement x Group: with surgery	0.358	0.304, 0.409	<0.001
**Diaphragmatic aorta**	Age at measurement	0.072	0.045, 0.100	<0.001
	Group: with surgery	-6.614	-8.392, -4.777	<0.001
	Antihypertensive medication: yes	-0.057	-0.672, 0.551	0.855
	Sex: male	0.782	-0.138, 1.696	0.098
	BMI	0.038	-0.014, 0.092	0.147
	Age at measurement x Group: with surgery	0.195	0.145, 0.243	<0.001
**Abdominal aorta**	Age at measurement	0.073	0.047, 0.101	<0.001
	Group: with surgery	-5.757	7.566, -3.861	<0.001
	Antihypertensive medication: yes	-0.010	-0.667, 0.643	0.976
	Sex: male	0.409	-0.492, 1.304	0.374
	BMI	0.037	-0.018, 0.094	0.188
	Age at measurement x Group: with surgery	0.171	0.122, 0.216	<0.001
